# Expression of S100A8 protein on B cells is associated with disease activity in patients with systemic lupus erythematosus

**DOI:** 10.1186/s13075-023-03057-z

**Published:** 2023-05-10

**Authors:** Koji Kitagori, Takuma Oku, Masaki Wakabayashi, Tomoya Nakajima, Ran Nakashima, Kosaku Murakami, Yoshitaka Hirayama, Yasushi Ishihama, Koichiro Ohmura, Akio Morinobu, Tsuneyo Mimori, Hajime Yoshifuji

**Affiliations:** 1 Department of Rheumatology and Clinical Immunology, Graduate School of Medicine, Kyoto University, Kyoto, Japan; 2 Center for Innovation in Immunoregulative Technology and Therapeutics, Graduate School of Medicine, Kyoto University, Kyoto, Japan; 3 Research Portfolio & Science, Astellas Pharma Inc, Tokyo, Japan; 4 Omics Research Center, National Cerebral and Cardiovascular Center, Osaka, Japan; 5 Department of Molecular and Cellular Bioanalysis, Graduate School of Pharmaceutical Sciences, Kyoto University, Kyoto, Japan; 6 Kobe City Medical Center General Hospital, Hyogo, Japan; 7 Ijinkai Takeda General Hospital, Kyoto, Japan

**Keywords:** Systemic lupus erythematosus, B cells, S100A8

## Abstract

**Background:**

Systemic lupus erythematosus (SLE) is an intractable disease characterized by autoantibody production and autoreactive B and T cell proliferation. Although several studies have revealed multiple genetic and environmental associations, the underlying mechanisms remain unknown.

**Methods:**

We performed proteomics and transcriptomics using liquid chromatography-mass spectrometry and DNA microarray, using peripheral blood B cells from patients with SLE, and healthy controls (HC). We explored molecules associated with the pathophysiology of SLE by flow cytometry and B cell stimulation assay.

**Results:**

We identified for the first time that expression of both S100A8 protein and mRNA were markedly upregulated in SLE B cells. The results obtained using flow cytometry showed that S100A8 was highly expressed on the surface of B cells of patients with active SLE (MFI; HC 102.5 ± 5.97, stable SLE 111.4 ± 12.87, active SLE 586.9 ± 142.9), and S100A8 on the cell surface was decreased after treatment (MFI; pre-treat 1094.5 ± 355.38, post-treat 492.25 ± 247.39); therefore, it is suggested that S100A8 may be a marker for disease activity. The mRNA expression of S100A8 was particularly upregulated in memory B cells of SLE (56.68 fold higher than HC), suggesting that S100A8 may be mainly secreted by memory B cells in the pathogenesis of SLE.

**Conclusions:**

Our results imply that the S100A8 proteins secreted from memory B cells may stimulate granulocytes and monocytes through pattern recognition receptors, activate the innate immune system, and are involved in the pathogenesis of SLE.

**Supplementary Information:**

The online version contains supplementary material available at 10.1186/s13075-023-03057-z.

## Background

Systemic lupus erythematosus (SLE) is a systemic autoimmune disease characterized by various immune abnormalities, including the appearance of autoreactive T and B cells [1], production of autoantibodies, and various organ disorders. Corticosteroids and various immunosuppressive agents are used as therapeutic interventions for SLE [2], but their side effects, such as compromised immune status and metabolic disorders, can cause serious problems. To administer appropriate drug dosages, disease activity markers are needed, along with safer and more effective treatments.

Autoreactive B cells contribute to SLE pathophysiology. The anti-B lymphocyte stimulator antibody showed significant therapeutic effects on SLE [3–5] and was approved by the Food and Drug Administration of the USA as a novel therapeutic agent for treating SLE in 2011. However, it remains unclear how B cell dysfunction is involved in the pathogenesis of SLE. Currently, it is difficult to isolate autoreactive lymphocytes. Establishing such a technique will lead to the development of more effective treatments with fewer side effects.

In recent years, various comprehensive analyses have been performed to detect molecules associated with the pathophysiology of autoimmune diseases, such as analysis of single nucleotide polymorphisms using genome-wide association studies (genomics), analysis of mRNA using next-generation sequencing and DNA microarray assays (transcriptomics), and analysis of proteins (proteomics) and metabolites (metabolomics) using mass spectrometry. Data from these multifarious analyses will enable the discovery of new molecules or pathological conditions. In the present study, we explored the pathophysiology of SLE using these methods.

We performed proteomics using liquid chromatography-mass spectrometry (LC–MS/MS) and transcriptomics using DNA microarray using peripheral blood B cells from patients with SLE and healthy controls. We tested molecules with high protein and mRNA expression levels in patients with SLE.

## Methods

### Participants

The study was approved by the Kyoto University Medical Ethics Committee (G488, G1172). We obtained written informed consent from all participants and collected blood samples (30 ml) from healthy controls (HC) and patients with SLE and other patients with connective tissue diseases (CTDs) who visited the Department of Rheumatology and Clinical Immunology at Kyoto University Hospital, and clinical information from their medical records.

We separated total B cells (CD19 +) from the peripheral blood of patients with SLE (*N* = 14) and HC (*N* = 10) (Supplementary Tables 1 and 2) and analyzed protein expression using LC–MS/MS. We separated total B cells (CD19 +), naïve B cells (CD19 + , CD27 −), memory B cells (CD19 + , CD27 +), T cells (CD3 +), monocytes (CD14 +), plasmacytoid dendritic cells (pDC; CD304 +), and myeloid dendritic cells (mDC; CD1c +) from the peripheral blood of patients with SLE (*N* = 7) and HC (*N* = 10) (Supplementary Tables 1 and 2), and analyzed RNA expression using DNA microarray. After we identified S100A8, we collected blood samples from HC (*N* = 18) and patients with SLE (*N* = 33) and performed verification using quantitative reverse transcription PCR (RT-PCR), flow cytometry (FCM), immunohistochemistry, and culture assays. The disease activity of each patient with SLE was evaluated using SLE Disease Activity Index 2000 (SLEDAI-2 K) [6], and the patients were divided into active (SLEDAI ≥ 6, *N* = 16), and stable (SLEDAI ≤ 5, *N* = 17) groups.

### Cell isolation

Peripheral blood mononuclear cells (PBMCs) were separated using Ficoll. We isolated 3–6 × 10^7^ of the PBMCs from each patient and control. We used 1 × 10^7^ of the PBMCs for the isolation of B cells with negative selection by magnetic-activated cell sorting (MACS; B Cell Isolation Kit II human, Miltenyi Biotec; CA, USA) or EasySep (EasySepTM Human B Cell Enrichment Kit, STEMCELL Technologies Inc., Vancouver, Canada). The separated B cells (mean purity: 97%) were used for the total B cell studies, as described below. The remaining PBMCs were divided into two groups: one group (2–5 × 10^7^ cells) was used to separate memory B cells (CD19 + CD27 +), naïve B cells (CD19 + CD27 −), pDCs (CD304 +), and mDCs (CD1c +) by BD FACSAria III, and the other (1 × 10^6^ cells) to separate monocytes and CD3 + T cells by MACS positive selection (EasySep Human CD14 Positive Selection Kit II, STEMCELL Technologies Inc.; Vancouver, Canada.; EasySep Human CD3 Positive Selection Kit II, STEMCELL Technologies Inc.; Vancouver, Canada).

### Liquid chromatography-mass spectrometry (LC–MS/MS)

CD19-positive cells (1 × 10^5^ cells/run) were analyzed using LC–MS/MS using a monolith column, in collaboration with Prof. Ishihama (Faculty of Pharmacy, Kyoto University). The exponentially modified protein abundance index (emPAI) value was calculated for each protein detected [7], and the expression levels of each were compared between the HC and patients with SLE.

$$\mathrm{emPAI }= {10}^{\mathrm{PAI}}-1$$

$$\mathrm{PAI }= (\mathrm{number\,of\,observed\,peptides\,per\,protein})/(\mathrm{number\,of\,observable\,peptides\,per\,protein})$$

### Flow cytometry (FCM)

To detect S100A8-positive cells, whole blood of HC and patients with SLE were stained with specific antibodies against CD19-V500 (clone HIB19, BD Biosciences, Franklin Lakes, NJ, USA), CD3-BV421 (clone OKT3, BD Biosciences, Franklin Lakes, NJ, USA), CD14-BUV395 (clone M5E2, BD Biosciences, Franklin Lakes, NJ, USA), CD11c-V450 (clone B-ly6, BD Biosciences, Franklin Lakes, NJ, USA), CXCR5-APC (clone #51,505, R&D Systems, NE Minneapolis, MN, USA), CD27-PE-Cy7 (clone O323, Thermo Fisher Scientific, Waltham, MA, USA), IgD-Horizon V500 (clone IA6-2, BD Biosciences, Franklin Lakes, NJ, USA), S100A8/A9-FITC, and S100A8-FITC and analyzed using BD LSRFortessa™. Antibodies specific to the S100A8/A9 complex (clone 27E10, BMA Biomedicals, Rheinstrasse, Switzerland) and S100A8 (clone 3H2617, Life Span BioSciences, Huissen, Netherlands) were used. The relative expression levels of each molecule were compared by mean fluorescence intensity (MFI).

### Enzyme-linked immunosorbent assay (ELISA)

The concentrations of S100A8 and S100A8/A9 in the plasma and supernatants were measured using MRP8 (S100A8) enzyme immunoassay (BMA Biomedicals, Rheinstrasse, Switzerland) and MRP8/14 (calprotectin) enzyme immunoassay (BMA Biomedicals, Rheinstrasse, Switzerland), respectively.

### Immunohistochemistry

We separated CD19-positive cells using negative selection using EasySep, prepared slides by cytocentrifugation, fixed them with 4% paraformaldehyde, and stained them with CD19-AlexaFluor647 (clone HIB19, BioLegend; San Diego, CA, USA), S100A8-FITC (clone 3H2617), and S100A8/A9-FITC (clone 27E10) antibodies (Cell Signaling Technology; https://www.cellsignal.jp/learn-and-support/protocols). The cells were observed using a confocal microscope (LSM 710; Zeiss).

### mRNA analysis

DNA microarray: Total RNA was extracted from each cell population using (RNAqueous™ -Micro Total RNA Isolation Kit, Invitrogen, Thermo Fisher Scientific; Waltham, MA, USA). The extracted RNA had an RNA integrity number of ≥ 7. cDNA (6.0 ng) was analyzed using a DNA microarray (SurePrint G3 Human Gene Expression Microarray 8 × 60 k V2.0; Agilent technology; Santa Clara, CA, USA) with 50,599 probes. We uploaded the data from the DNA microarray analysis to National Center for Biotechnology Information in 2020 (GSE: 148,601).

Quantitative PCR: We analyzed 0.2–10.0 ng of the cDNA of memory, naïve, and total B cells using the Fluidigm system (Fluidigm, South San Francisco, CA, USA). We performed TaqMan assays (Thermo Fisher Scientific, Inc.; Waltham, MA USA) to quantify the gene expression level using 1.0 ng of cDNA. *ACTB* was used as the housekeeping gene.

The following PCR primers were used for qPCR: S100A8, GCCAAGCCTAACCGCTATAA and ATGATGCCCACGGACTTG; S100A9, GTGCGAAAAGATCTGCAAAA and TCAGCTGCTTGTCTGCATTT; LTF, GTGTCCAGGCTGACAGAAGTT and CGCACCACTGAACACTCCT; RETN, CCATGGAAGAAGCCATCAA and CTGGCAGTGACATGTGGTCT; NFATC1, CCAAGGTCATTTTCGTGGAG and GGTCAGTTTTCGCTTCCATC; DEFA3, CCTGCCTAGCTAGAGGATCTGT and CATCAGCTCTTGCCTGGAGT; PAD4, AACCAGAGCTGTGAAAGATCAGA and TCACAGTTCACCAGCAGGAT; LCN2, CAGGACTCCACCTCAGACCT and CCAGGCCTACCACATACCAC; CTSG, TCTGCTGGCCTTTCTCCTAC and GGATCTGAAGATACGCCATGT, and CAPM, TCGGATGCTAACCTCTACCG and ACAGGCTTTGGCGTGTCT.

### B cell stimulation

We isolated B cells from the peripheral blood of HC and patients with SLE using negative selection. The cells were then suspended in an RPMI medium containing 10% fetal calf serum at a concentration of 1 × 10^5^ cells/200 μl. Various stimulatory molecules were added to each culture medium, including anti-IgG/IgM antibody (25 ug/ml) for B cell receptor (BCR) stimulation, LPS (20 ug/ml) for toll-like receptor (TLR) 4 stimulation, imiquimod (10 ug/ml) for TLR7 stimulation, and CpG (4 ug/ml) for TLR9 stimulation. Further, CD40L (5 ug/ml) and IL-4 (20 ng/ml) were used as maintenance additives. We also used phorbol 12-myristate 13-acetate (PMA, 20 ng/ml) and ionomycin (1 μM) as positive controls. We additionally performed BCR stimulation with IFN-α (1000U/ml) on B cells from HC. The cells were then cultured at 37 °C in 5% CO_2_ for 24 h. The concentrations of S100A8 and S100A8/A9 in the supernatants were measured using ELISA.

### Statistical analysis

The Mann–Whitney *U* test was used to compare the gene expression levels and protein concentrations between the two groups. The Kruskal–Wallis test was used to compare three or more groups. A *p* value < 0.05 was considered to be significant.

### Database mining

We used the Ingenuity Pathway Analysis program (IPA; QIAGEN, Hilden, Germany) to determine which pathways included specific molecules that we had detected using DNA microarray and LC–MS/MS. We then used NextBio software (Illumina, San Diego, CA, USA) to determine the molecular functions of these target molecules.

We subtracted type I interferon (IFN)-related genes from our data using three datasets about treatment for HCV-related chronic hepatitis with IFN-α from NextBio, which analyzed genes of intracellular molecules that were differentially expressed before and after stimulation or treatment with type I IFN.

1. 158 genes from the dataset were genes involved in interferon signaling.
2. 294 genes from the dataset included PBMCs of patients with chronic HCV infection who had undergone 12 weeks of IFN-α and ribavirin therapy vs. untreated, and PBMCs from patients with multiple sclerosis after 3 months of treatment with betaferon vs. baseline).
3. 709 genes from the dataset were from PBMCs of patients with chronic HCV infection who had undergone 12 weeks of IFN-α and ribavirin therapy vs. untreated, and PBMCs from patients with multiple sclerosis after 3 months of treatment with Rebif® vs. baseline.

Overlapping molecules were excluded and 880 molecules were referenced as IFN signatures.

## Results

We enrolled 14 and 7 patients with SLE for proteomic and transcriptomic analyses, respectively (Supplementary Table 1). Supplementary Table 2 shows the treatment details of the patients. We grouped patients with SLE into active or stable states according to their SLEDAI scores.

We comprehensively searched for molecules that were differentially expressed in SLE B cells by DNA microarray and proteomics. The top 10 molecules with the greatest increase in expression level in each analysis of CD19 + B cells in patients with SLE compared to those in HC are shown in Supplementary Table 3, in descending order of fold change. In the previous reports, type 1 IFN was associated with the pathogenesis of SLE [8], however, we considered that pathogenesis different from IFNs was of interest. Consistent with previous reports, this list contains many IFN-related genes. We subtracted the IFN-related genes using the NextBio datasets (see Methods). Table 1 shows the 10 most differentially expressed molecules in patients with SLE after subtraction. We examined those molecules which are not related to IFN signals and related to SLE pathology. Next, we analyzed the functions and roles of the selected molecules using pathway analysis (IPA). The top five pathways listed were (1) glucocorticoid receptor signaling, (2) production of nitric oxide and reactive oxygen species in macrophages, (3) clathrin-mediated endocytosis signaling, (4) oxidative phosphorylation, and (5) interleukin (IL)-12 signaling and production in macrophages.

**Table 1:** Molecules with the greatest expression after the removal of IFN-related molecules

Proteomics				Transcriptomics		
Rank	Molecule	Fold change	*P*	Molecule	Fold change	*P*
1	HSPB1	4.379	0.038	DEFA3	315.044	0.008
2	SERPINC1	4.152	0.004	S100A8	35.312	0.001
3	HBA1 / HBA2	4.080	0.001	LTF	29.498	0.019
4	LTF	3.269	0.040	CAMP	18.392	0.040
5	C3	2.808	0.002	S100P	16.269	0.001
6	ITIH2	2.745	0.049	S100A9	15.451	0.005
7	ANP32B	2.657	0.000	CTSG	14.096	0.019
8	MNDA	2.646	0.022	CH25H	13.153	0.001
9	ALDH2	2.594	0.026	RETN	11.266	0.008
10	ITIH3	2.549	0.005	S100A12	10.451	0.025

Molecules that were significantly increased in patients with SLE compared to that in HC after the exclusion of IFN-related molecules in the proteomics and transcriptomics results. The 10 molecules with the greatest increase in expression by fold change are listed

**Fig. 1:**
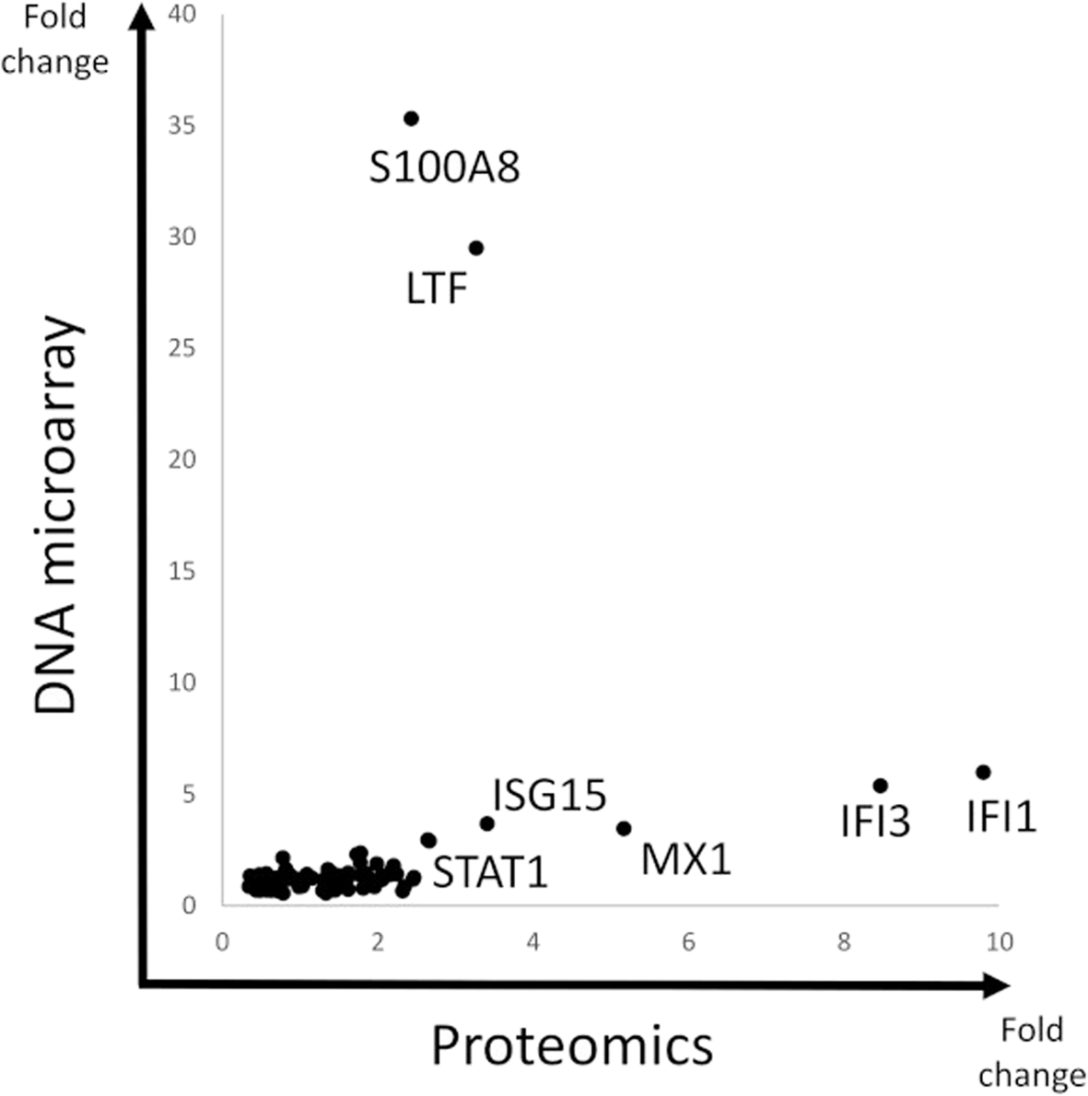
The molecules upregulated in SLE identified by proteomics and DNA microarrays. Each molecule is plotted as fold changes in proteomics and DNA microarrays

As shown in Table 1, hemoglobin (HBA1/HBA2), lactoferrin (LTF), complement (C3), defensin (DEFA3), S100 families, and cathelicidin antimicrobial peptides (CAMP/LL-37) were among the top 10 molecules with the greatest increase in expression in SLE B cells. The S100 family and LTF are usually expressed in myeloid cells, but we found them in B cells. We plotted each molecule by fold changes of proteomics and DNA microarray before subtraction of IFN-related molecules in Fig. 1. Most of the molecules were plotted on a linear line, and IFN-related genes (IFI1, IFI3, MX1, etc.) showed high fold changes. However, S100A8 and LTF showed the highest fold-changes in the DNA microarray analysis. Although the other S100 families are listed in Table 1, only S100A8 is shown in Fig. 1. This is because the protein expression of other S100 molecules was not detected using our proteomics method with emPAI. Additionally, we also performed cluster analysis with DNA microarray data and found that each S100A8 and IFN-α was classified into different clusters (data not shown). Therefore, we focused on S100A8 and its related molecules.

The S100A8 is known as a molecule secreted from neutrophils and monocytes. Therefore, we analyzed S100A8 mRNA expression levels not only in lymphocytes but also monocytes and dendritic cells that are considered to be related to SLE pathogenesis. We sorted total B cells, memory B cells, naïve B cells, T cells, mDCs, and pDCs from the peripheral blood of patients with SLE and HC and performed DNA microarray analysis. The genes with expression levels highly correlated with S100A8 in the total B cells are listed in Supplementary Table 4, with the majority being expressed in myeloid cells. The expression pattern of the genes in total B cells was like that in memory B cells (CD19 + /CD27 +) and mDCs. Next, S100A8 and seven related genes were selected, and their expression was validated using quantitative RT-PCR (Supplementary Table 5). Memory B cells showed higher FCs than did naïve B cells, and the gene expression pattern of memory B cells was like that of the total B cells.

**Fig. 2:**
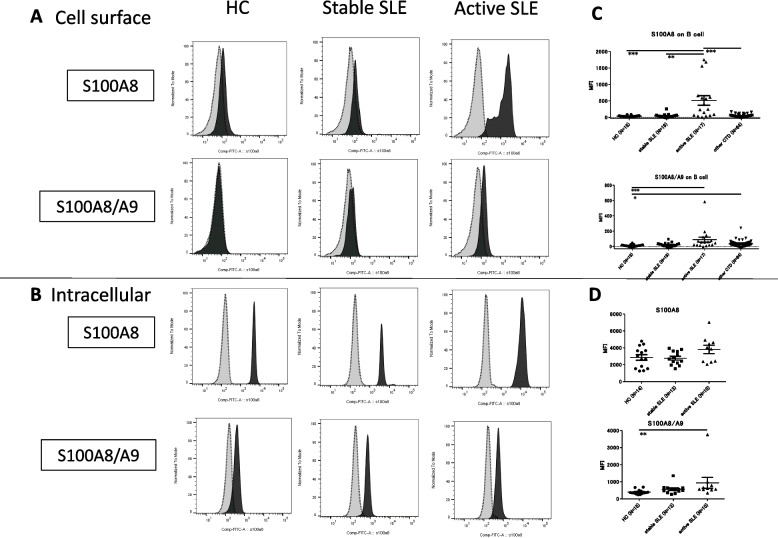
**A** The expression levels of S100A8 and S100A8/A9 on the cell surface and intracellular peripheral blood CD19 + cells of HC, stable SLE, and active SLE were evaluated using flow cytometry. S100A8 and S100A8/A9 on the cell surface of B cells in active SLE were significantly increased compared to those in HC. **B** In the intracellular staining, no significant difference was observed among the groups. **C**, **D** The cell surface (**C**) and intracellular (**D**) expression of S100A8 and S100A8/A9 were evaluated in various connective tissue diseases (CTDs); however, its expression was not as high as that in SLE. Active SLE was classified as having SLEDAI score ≥ 6. Statistical significance is denoted by asterisks: **, *p* < 0.01; ***, *p* < 0.001 in Kruskal–Wallis. Other CTDs include polyarteritis nodosa (*N* = 4), Takayasu’s arteritis (*N* = 8), eosinophilic granulomatosis with polyangiitis (*N* = 6), granulomatosis with poly angiitis (*N* = 2), microscopic polyangiitis (*N* = 5), polymyositis/dermatomyositis (*N* = 9), Behçet disease (*N* = 2), IgG4-related disease (*N* = 1), Sjögren syndrome (*N* = 5), relapsing polychondritis (*N* = 5), systemic sclerosis (*N* = 6), ankylosing spondylitis (*N* = 1), adult-onset Still’s disease (*N* = 1), and rheumatoid arthritis (*N* = 5)

**Fig. 3:**
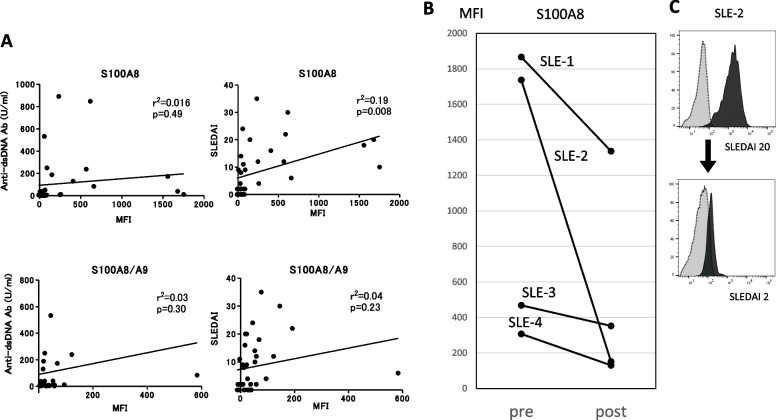
**A** Association of expression levels of S100A8 and S100A8/A9 on B cells with disease activity. The MFI of S100A8 on the B cell surface correlated with SLEDAI. We used monoclonal antibody 27E10 which recognizes S100A8/A9 dimers and monoclonal antibody 3H2617 which recognizes S100A8. **B** S100A8 on the surface of peripheral blood B cells was evaluated using flow cytometry in patients with SLE (*N* = 4) whose blood samples were obtained before and after treatment (initial or strengthened therapy). In all four patients, S100A8 expression was significantly reduced after treatment. **C** The change of anti-S100A8 staining patterns before and after the treatment in one patient with SLE (SLE-2)

S100A8 is typically expressed as a monomer, homodimer, or heterodimer with S100A9. To validate the elevated expression of S100A8 protein in SLE B cells, we analyzed the expression of S100A8 and S100A8/A9 proteins using FCM (Fig. 2A). We used two types of monoclonal antibodies: (1) a monoclonal antibody that recognizes only S100A8 (3H2617) and (2) a monoclonal antibody that specifically recognizes the S100A8/A9 heterodimer (27E10). The expression levels of S100A8 and S100A8/A9 were significantly higher on the cell surface of CD19^+^ B cells in patients with SLE than in HC, especially only in those in the active group. Next, intracellular staining was performed using monoclonal antibodies. Both S100A8 and S100A8/A9 were shown to be present in the B cells. However, there was no significant difference in the intracellular expression levels of S100A8 and 100A8/A9 between patients with SLE and HC (Fig. 2B). Next, we investigated the correlation between the expression levels (MFI) of S100A8 and S100A8/A9 on the surface of CD19^+^ B cells, SLEDAI-2 K, and serum anti-dsDNA antibody titer. There was a significant correlation between S100A8 MFI and SLEDAI-2 K scores (*r*^2^ = 0.19, *p* = 0.008) (Fig. 3A). We also evaluated S100A8 expression on double negative (DN)-2 B cells (CD19^+^, CD27^−^, CXCR5^−^, IgD^−^, CD11c^+^), which has recently been reported to be associated with SLE pathology [9]. As a result, there was no significant difference in surface S100A8 expression level between memory B cells and DN2 B cells (Supplementary Fig. 3). It implied that the increase of S100A8 on the surface of SLE B cells was not specific to DN2 B cells. Further, we analyzed S100A8 expression levels on the surface of peripheral B cells from patients with SLE (*N* = 4) who had donated blood samples before and after treatment (initial or strengthened therapy). In all cases, the expression level of S100A8 on the surface of peripheral blood B cells decreased after treatment (Fig. 3B and C).

**Fig. 4:**
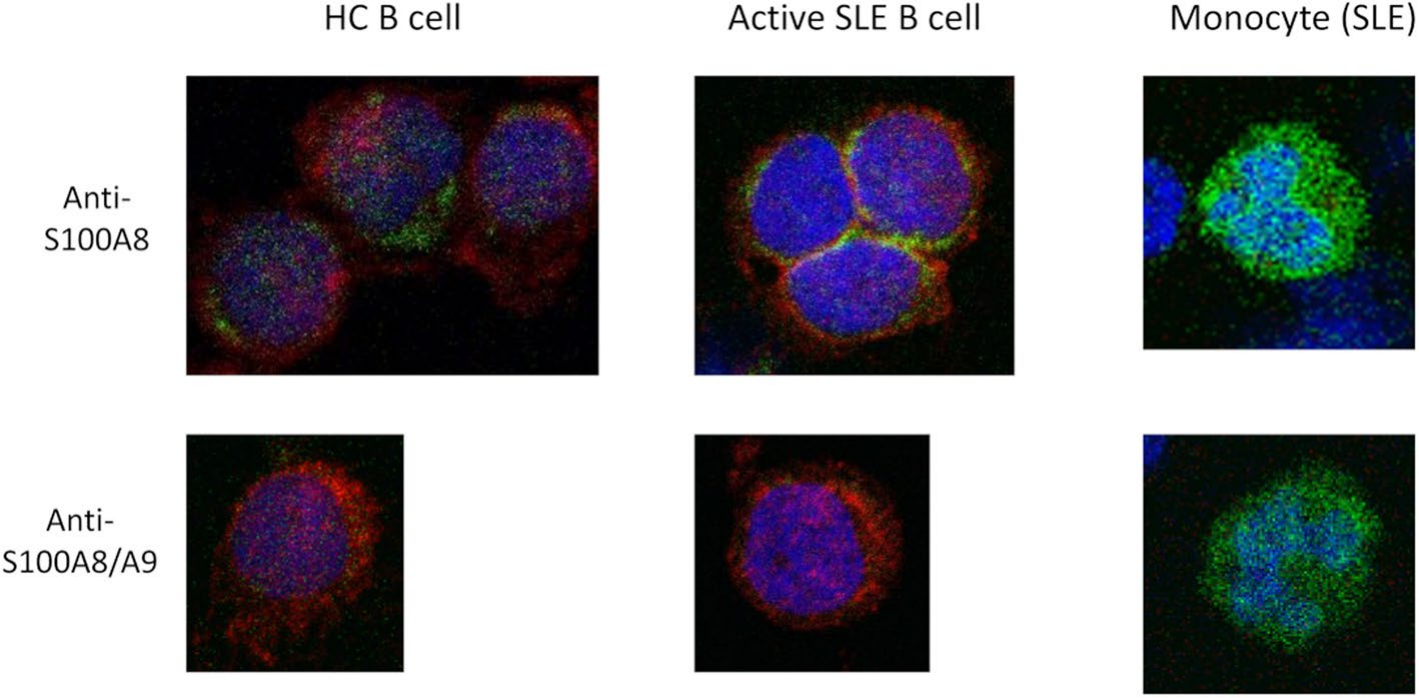
Analysis of S100A8 and S100A8/A9 expression and localization in peripheral blood B cells using confocal microscopy. Peripheral B cells were fixed and stained by antibodies against CD19 (red), nucleus (blue), and S100A8 or A8/A9 (green) by confocal microscopy. In the anti-S100A8 (3H2617) staining, the rims of B cells from patients with active SLE were stained, while B cells from the HC were not stained. The cytoplasm of monocytes was brightly stained by 3H2617, acting as the positive controls. Anti-S100A8/A9 (27E10) did not stain the B cells of patients with SLE or HC

We also analyzed the expression and the localization of S100A8 in B cells using confocal microscopy (Fig. 4). In staining with anti-S100A8 (3H2617), the rims of B cells from a patient with active SLE were stained, while B cells from the HC were only slightly stained. Anti-S100A8/A9 (27E10) did not stain B cells in patients with SLE or HC.

We measured plasma S100A8 and S100A8/A9 protein concentrations and compared them between patients with SLE and HC. The plasma S100A8/A9 protein concentration was significantly higher in patients with SLE than in HC (Supplementary Fig. 1). Plasma S100A8 protein concentration in patients with SLE showed a similar tendency but did not show a significant difference when compared with that in the HC group.

**Fig. 5:**
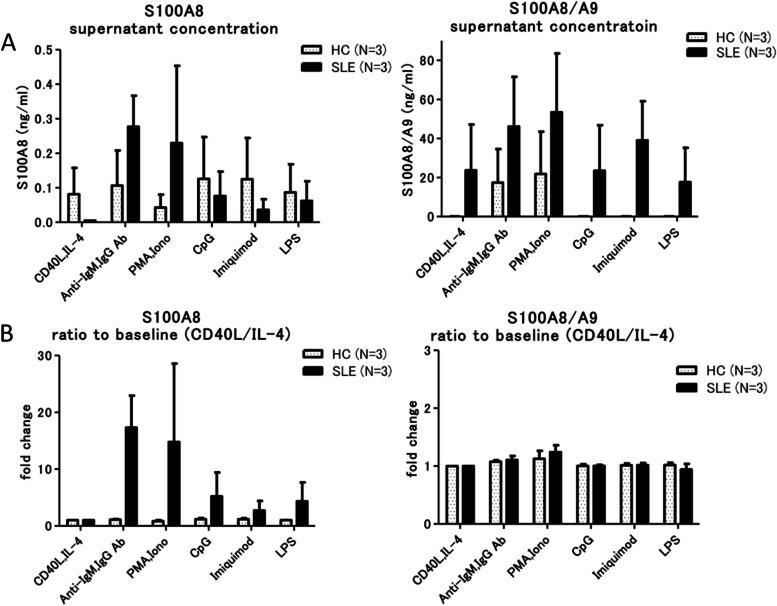
B cells from the patients with SLE and HC were cultured with maintenance additives (CD40L and IL-4), and the supernatant concentration of S100A8 and S100A8/A9 was measured after stimulation with anti-IgM/IgG, PMA/ionomycin, CpG, imiquimod, and LPS. **A** The supernatant concentration of S100A8 from the B cells of patients with SLE was elevated compared to those of HC after the stimulation by anti-IgM/IgG and PMA/ionomycin. The concentration of S100A8/A9 was elevated by all the stimuli. **B** The ratios of the concentration to the baseline (CD40L and IL-4 only) are indicated. The ratio of S100A8 from the B cells of patients with SLE was markedly elevated when stimulated by anti-IgM/IgG and PMA/ionomycin

We hypothesized that S100A8 is released from B cells. Peripheral blood B cells of patients with SLE and HC were isolated and cultured in vitro and stimulated with various compounds, and S100A8 and S100A8/A9 protein concentrations were measured in the supernatant (Fig. 5). When stimulated by anti-IgG/IgM antibodies or PMA/ionomycin, the concentration of S100A8 was markedly elevated in the supernatant of B cells from patients with SLE compared with that of B cells from HC. We also stimulate the B cells from HC with IFN-α and anti-IgG/IgM antibodies and measured S100A8 in supernatant. As a result, S100A8 concentration was not elevated in those stimulations (Supplementary Fig. 4). Then, we investigated the expression levels (MFI) of S100A8 and S100A8/A9 on the surface of B cells after anti-IgG/IgM stimulation. The expression of S100A8 on the surface of B cells was not increased by the stimuli in both SLE and HC by FCM.

To examine the specificity of S100A8 and S100A8/A9 for SLE, their expression on the surface of B cells from patients with various connective tissue diseases was analyzed using FCM but was not as high as that in patients with active SLE (Fig. 2C and D).

## Discussion

In the present study, we performed proteomics (LC–MS/MS) and transcriptomics (DNA microarray) of B cells in patients with SLE and HC and tested for molecules that are differentially expressed in SLE. As previously reported, the activation of IFN-related molecules and related pathways was upregulated in patients with SLE [10]. In order to understand the pathology of SLE, we considered that an analysis different from the previous reports was of interest. We searched for molecules that are not associated with IFN but are associated with SLE disease activity. After excluding IFN-related molecules using the NextBio database, we focused on S100A8 and related molecules. Considering the high purity of CD19 + cells (> 95%) in the present study, granulocyte contamination should be minimal. The expression levels of S100A8 and S100A9 were enhanced, especially in the memory B cells of patients with SLE. Furthermore, the upregulation of S100A8 and S100A8/A9 proteins in peripheral blood B cells was rarely detected in patients with other autoimmune diseases. Therefore, an increase in S100A8 gene expression in B cells is considered specific for SLE. We investigated the association between S100A8 and the pathophysiology of SLE.

S100A8 protein predominately forms the S100A8/A9 complex and is usually present in granulocytes, such as neutrophils and macrophages [11–15]. The S100A8/A9 complex is a damage-associated molecular pattern that can act as a ligand for TLR4 and receptor for advanced glycation endproducts (RAGE), activate innate immune responses, and exhibit antibacterial activity [16–19]. The serum S100A8/A9 complex has been shown to be associated with disease activity in inflammatory diseases such as rheumatoid arthritis [20, 21], adult-onset Still’s disease [22], Sjögren’s syndrome [23], ankylosing spondylitis [24], Crohn’s disease [25], and ulcerative colitis [26]. In patients with SLE, serum S100A8/A9 and S100A12 levels were correlated with disease activity [27]; however, they were considered to be secreted from granulocytes in the peripheral blood. We also confirmed protein expression in SLE B cells by FCM and immunohistochemistry. It is unknown why anti-S100A8/A9 staining (27E10 antibody) did not work well in immunohistochemistry. We hypothesized that it might be due to fixation problems or differences in the sensitivity between FCM and immunohistochemistry because the same antibody worked intracellularly and on the cell surface in FCM. Our finding that S100A8 is highly expressed in B cells from the peripheral blood of patients with active SLE has not previously been reported.

As the upregulated expression level of S100A8 mRNA in SLE B cells disproportionally exceeded the upregulated S100A8 protein level (Fig. 1), it is speculated that S100A8 was secreted extracellularly. In our in vitro study, the supernatant S100A8 concentration from B cells of patients with SLE was markedly elevated after BCR stimulation compared to that of HC (Fig. 5). Type 1 IFN is latently elevated in SLE patients, therefore, stimulation of HC B cells with BCR alone may not adequately reflect the state of SLE B cells. Then, we performed an additional experiment in B cell stimulation assay. We added IFN-α and BCR stimulation in HC B cells. As a result, HC B cells did not show the S100A8 secretory response as SLE B cells. While S100A8/A9 is secreted in response to various stimuli, S100A8 is secreted specifically by BCR stimulation in SLE. Therefore, it is necessary to analyze more cases.

Based on the results of the present study, we suggest a hypothetical schema (Supplementary Fig. 2) for the role of S100A8 in the pathophysiology of SLE. S100A8 and S100A8/A9 activate macrophages and neutrophils by binding to their receptors (TLR4 and RAGE) and stimulate the innate immune system, leading to activation of the acquired immune system. As a result, new autoreactive B cells differentiate and mature, and these memory B cells release additional S100A8 and S100A8/A9, forming an inflammatory cycle by innate immune system activation. In our plasma analysis (Supplementary Fig. 1), plasma S100A8/A9 levels were significantly higher in patients with SLE than in HC. There was no statistically significant difference in plasma S100A8 (monomer or homodimer) concentrations among HC, active, and stable SLE. As the concentration of S100A8/A9 was approximately 3000 ng/ml, while the concentration of S100A8 was approximately 3 ng/ml, it is considered that the S100A8 mainly causes local inflammation and triggers the activation of innate immunity. The S100A8/A9 heterodimer is considered to be the main factor of the systemic inflammation. As S100A8 was predominantly highly expressed on the surface of B cells in patients with active SLE (Fig. 2), S100A8 (monomer or homodimer) is considered to be associated with B cell activation and is an igniter of innate immune activation triggered by BCR stimulation. However, there is a limitation that we did not exclude the possibility for alternative sources of serum S100A8, such as monocytes and neutrophils.

S100A8 on the B cell surface can be utilized as a marker of SLE disease activity. In the present study, upregulation of S100A8 was observed in the peripheral blood B cells of patients with active SLE, and the expression level of S100A8 on the B cell surface of SLE was correlated with SLEDAI scores. There is a limitation we did not verify the relationship between S100A8 and IFNs directly. However, cluster analysis with DNA microarray data showed that S100A8 and IFN-related molecules are classified into different clusters. Therefore, S100A8 on the surface of B cells may also be a new therapeutic target for selectively suppressing activated B cells in active SLE. We speculate that S100A8 released from SLE B cells may bind to the surface of B cells as a monomer or dimer, or form a heterodimer with S100A9 and circulate in the plasma. However, it was not clarified in the present study whether the scaffold of S100A8 on the cell surface is a receptor or another molecule. Further studies are required to confirm this hypothesis.

Table 1 includes LTF, CAMP/LL-37, and DEFA3, which have been previously reported as a neutrophil extracellular trap (NETs)-related molecules [28]. In the present study, the expression levels of these molecules strongly correlated with S100A8 levels. NETosis is the antibacterial function of neutrophils that releases intracellular proteins and nucleic acids. The production of autoantibodies against these intracellular components may be involved in the pathophysiology of SLE [29]. In recent years, similar reactions by eosinophils [30], pDCs [31], and lymphocytes [32] have been reported. Rocha et al. reported ETosis of B and T cells in patients with SLE [32]. Thus, ETosis may play a role in the pathogenesis of SLE. The upregulated expression of NET-related molecules in B cells in the present study may be associated with the ETosis of B cells. Therefore, further investigations are required.

## Conclusions

In a comprehensive analysis focusing on the B cells of patients with SLE, we found that S100A8 is highly expressed in the B cells of patients with active SLE. We confirmed that S100A8 is present on the B cell surface of SLE and is released from B cells upon BCR stimulation. The cell surface expression of S100A8 is specific for SLE, correlated with SLEDAI-2 K, and suppressed after treatment, suggesting that it may be a marker of SLE disease activity. S100A8 may be involved in the pathophysiology of SLE and serve as a target for new treatment options.

## Supplementary Information

**Additional file 1: Supplementary Table 1. **Profiles of the HC and SLE patients. Profiles of the HC and SLE patients enrolled in the proteomics (LC-MSMS) and transcriptomics (DNA microarray) analysis. Data are means ± S.D. HC: healthy controls, SLE: systemic lupus erythematosus, SLEDAI: SLE disease activity index.

**Additional file 2:** **Supplementary Table 2. **Detailed information of the registered patients. Detailed information of the SLE patients enrolled in the proteomics (LC-MSMS) and transcriptomics (DNA microarray) analysis. The samples of six patients were used for both proteomics and transcriptomics (SLE P1, P3, P5, P10, P11 and P14). *SLEDAI-2K ≥ 6 was defined as active. **Clinically diagnosed withoutrenal biopsy. Glucocorticoid dose was calculated as prednisolone-equivalent. AZA:azathioprine, MZB: mizoribine, RTX: rituximab.

**Additional file 3: Supplementary Table 3. **Top 10 before removal of IFN-related molecules.  The molecules that were significantly increased in SLE compared to HC in the analysis of proteomics and transcriptomics. The top 10 molecules by fold change were listed. Both lists contained many IFN-related molecules.

**Additional file 4: Supplementary Table 4.** The genes whose expression were highly correlated with S100A8 in B cells.  The genes whose expression were highly correlated with S100A8 in peripheral B cells were listed by Pearson’s correlation coefficients. The gene expression was analyzed by DNA microarray in the peripheral total B cells, memory B cells, Naïve B cells, T cells, monocytes, pDC, and mDC. Fold changes in the SLE patients compared with HC are shown. The listed molecules included many granulocyte-related molecules. The expression pattern in the total B cells was similar to those in memory B cells and mDC. FC: fold change, pDC: plasmacytoid dendritic cell, mDC: myeloid dendritic cell.

**Additional file 5: Supplementary Table 5. **Expression of S100A8 and its highly correlated genes by quantitative RT-PCR. The expression of S100A8 and seven genes that showed a high correlation with S100A8 in DNA microarray were validated by quantitative RT-PCR in the peripheral total B cells, memory B cells, and naïve B cells. The values are fold changes (FC) in the SLE patients compared with HC. Memory B cells showed higher FCs than naïve B cells. ND: Not detected both in the SLEpatients and HC, ∞: Not detected in the HC.

**Additional file 6: Supplementary Figure 1.** Concentration of S100A8 and S100A8/A9 in plasma. Plasma S100A8/A9 concentration was significantly higher in SLE.　Plasma S100A8 concentration was not significantly different between HC and SLE. Mann-Whitney U test as performed for comparison between two groups. Kruskal-Wallis test was performed for multiple comparison. *: *p*<0.05. **: *p*<0.01.

**Additional file 7: Supplementary Figure 2.** Hypothetical schema of the involvement of S100A8 and S100A8/A9 in the pathophysiology of SLE. S100A8 and S100A8/A9 produced and secreted by memory B cells stimulates neutrophils and monocytes through TLR4. The innate immune cells activate acquired immune cells (T cells and B cells) by antigen presentation. Autoimmunity was promoted by this inflammatory cycle.

**Additional file 8: Supplementary Figure 3.** Expression of S100A8 on DN2 B cells in SLE. In the peripheral blood of SLE patients, there was no significant change in the expression level of S100A8 on B cells between the memory B cell group and the DN2 B cell group.

**Additional file 9: Supplementary Figure 4. **Concentration of S100A8 in supernatant after stimulation on HC B cells with IFN-α and Anti-IgG/IgM. IFN-α and Anti-IgG/IgM were used to stimulate HC B cells. A. The supernatant concentration of S100A8 from the B cells of HC after the stimulation by IFN-α and/or Anti-IgG/IgM. The concentration of S100A8 showed no significant difference after the IFN-α andAnti-IgG/IgM stimulation. B. The ratios of the concentration to the baseline (CD40L and IL-4 only) are indicated. The ratio of S100A8 from the B cells of HC was not elevated after the stimulation.

## Data Availability

The datasets of DNA microarray generated during this study are available in National Center for Biotechnology Information (GSE: 148,601). The datasets of LC-MSMS generated during this study are not publicly available due to an intellectual property protection agreement with Astellas Pharma Inc.
