# Supplementary figures and images for: Expression of S100A8 protein on B cells is associated with disease activity in patients with systemic lupus erythematosus
Source: Arthritis Res Ther. 2023 May 10;25:76. doi: 10.1186/s13075-023-03057-z (PMC10170829; doi:10.1186/s13075-023-03057-z)

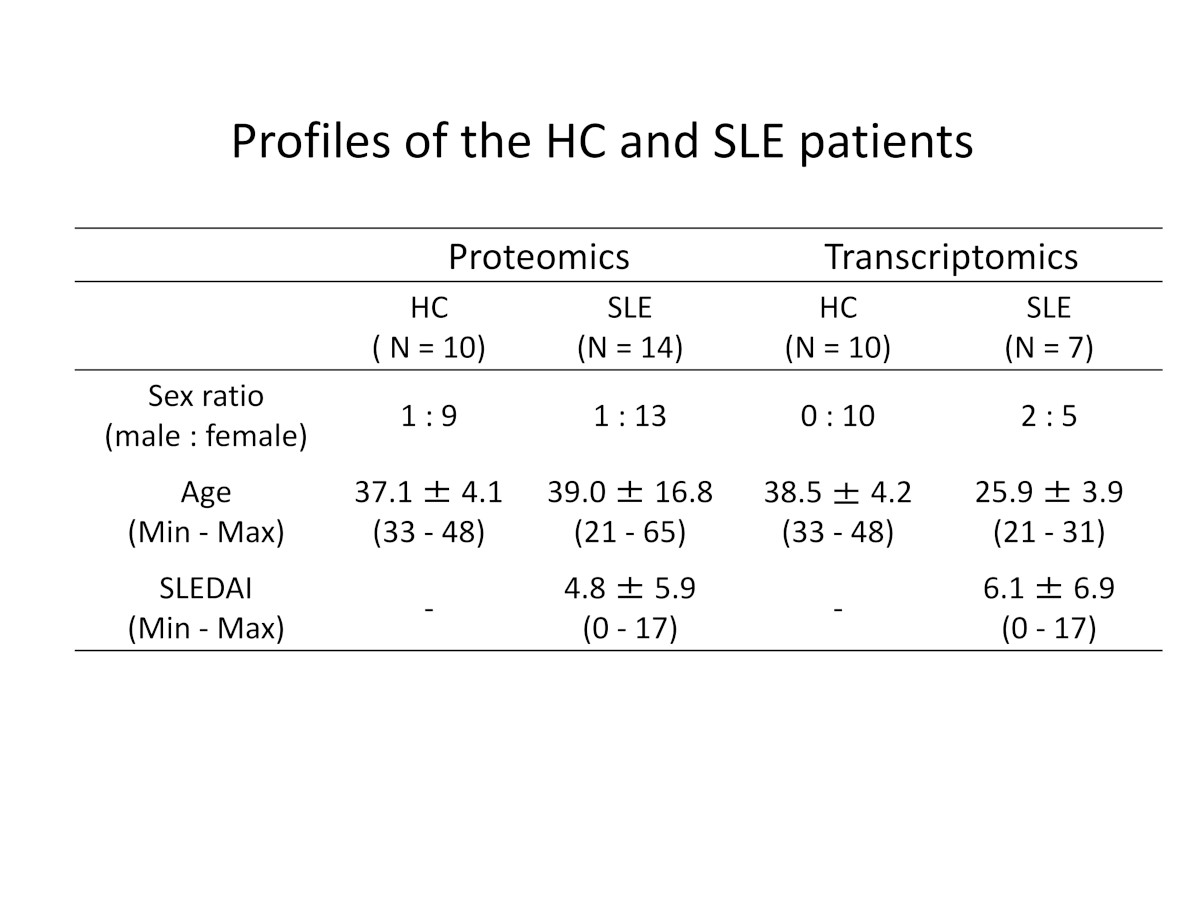

Supplement: Supplementary file 1 — Additional file 1: Supplementary Table 1. Profiles of the HC and SLE patients. Profiles of the HC and SLE patients enrolled in the proteomics (LC-MSMS) and transcriptomics (DNA microarray) analysis. Data are means ± S.D. HC: healthy controls, SLE: systemic lupus erythematosus, SLEDAI: SLE disease activity index. [file 13075_2023_3057_MOESM1_ESM.jpg]

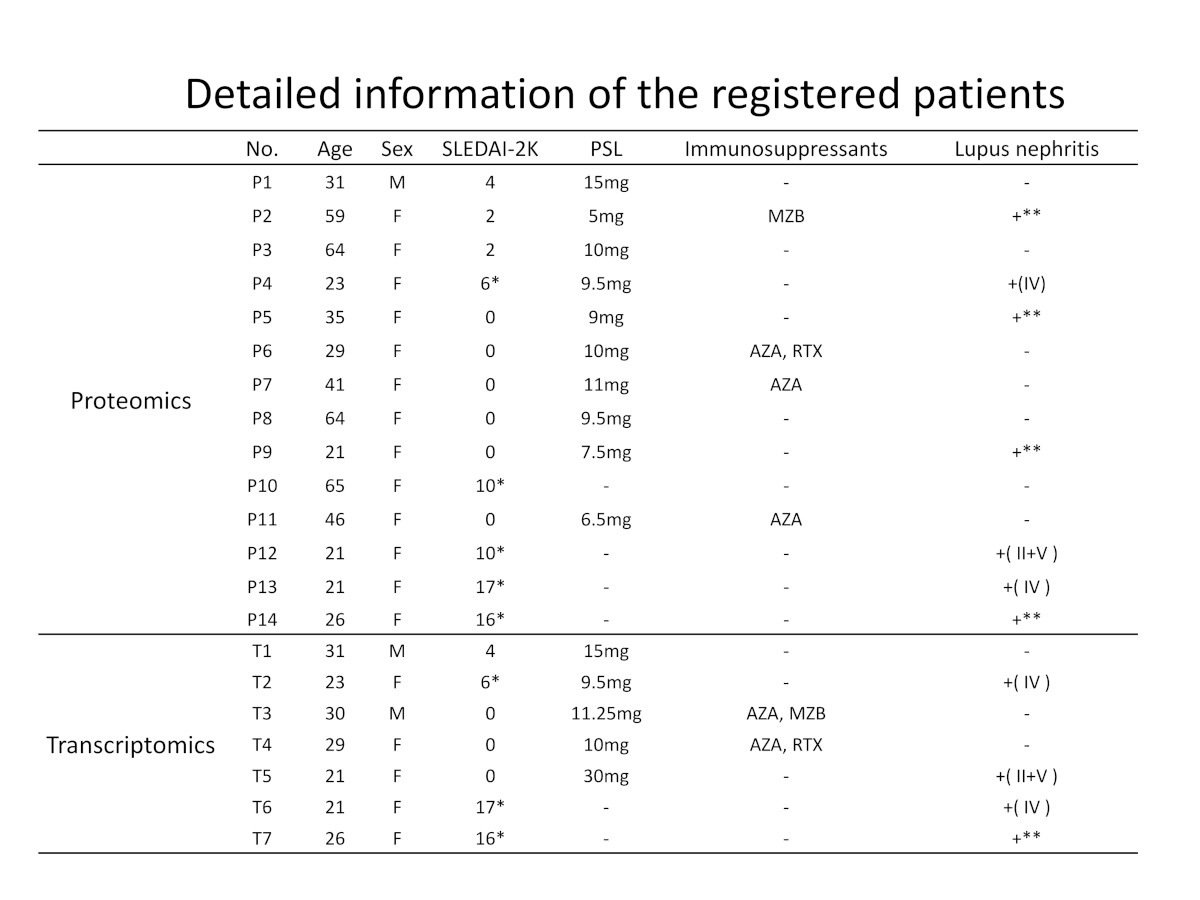

Supplement: Supplementary file 2 — Additional file 2: Supplementary Table 2. Detailed information of the registered patients. Detailed information of the SLE patients enrolled in the proteomics (LC-MSMS) and transcriptomics (DNA microarray) analysis. The samples of six patients were used for both proteomics and transcriptomics (SLE P1, P3, P5, P10, P11 and P14). *SLEDAI-2K ≥ 6 was defined as active. **Clinically diagnosed withoutrenal biopsy. Glucocorticoid dose was calculated as prednisolone-equivalent. AZA:azathioprine, MZB: mizoribine, RTX: rituximab. [file 13075_2023_3057_MOESM2_ESM.zip › 1-Supplementary table 2 S100 20230318.jpg]

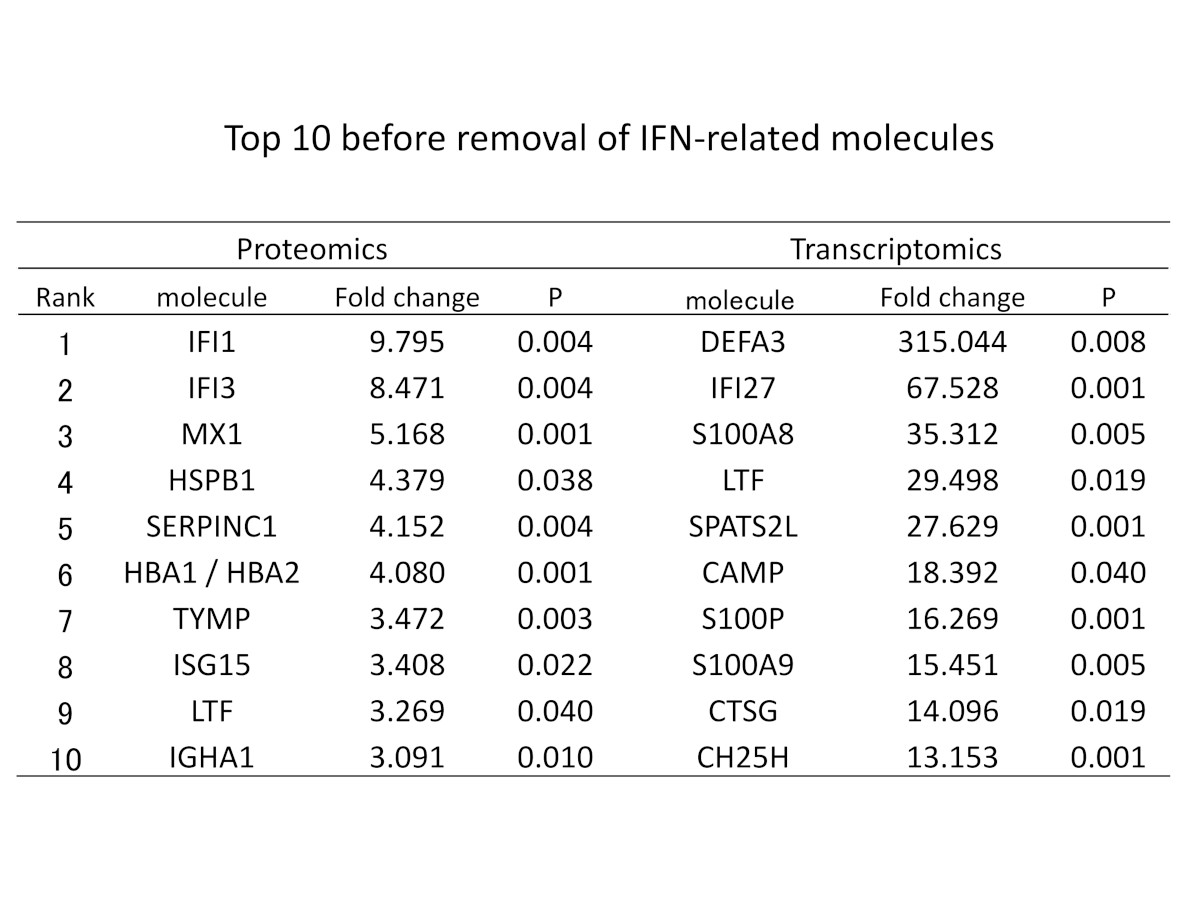

Supplement: Supplementary file 3 — Additional file 3: Supplementary Table 3. Top 10 before removal of IFN-related molecules. The molecules that were significantly increased in SLE compared to HC in the analysis of proteomics and transcriptomics. The top 10 molecules by fold change were listed. Both lists contained many IFN-related molecules. [file 13075_2023_3057_MOESM3_ESM.jpg]

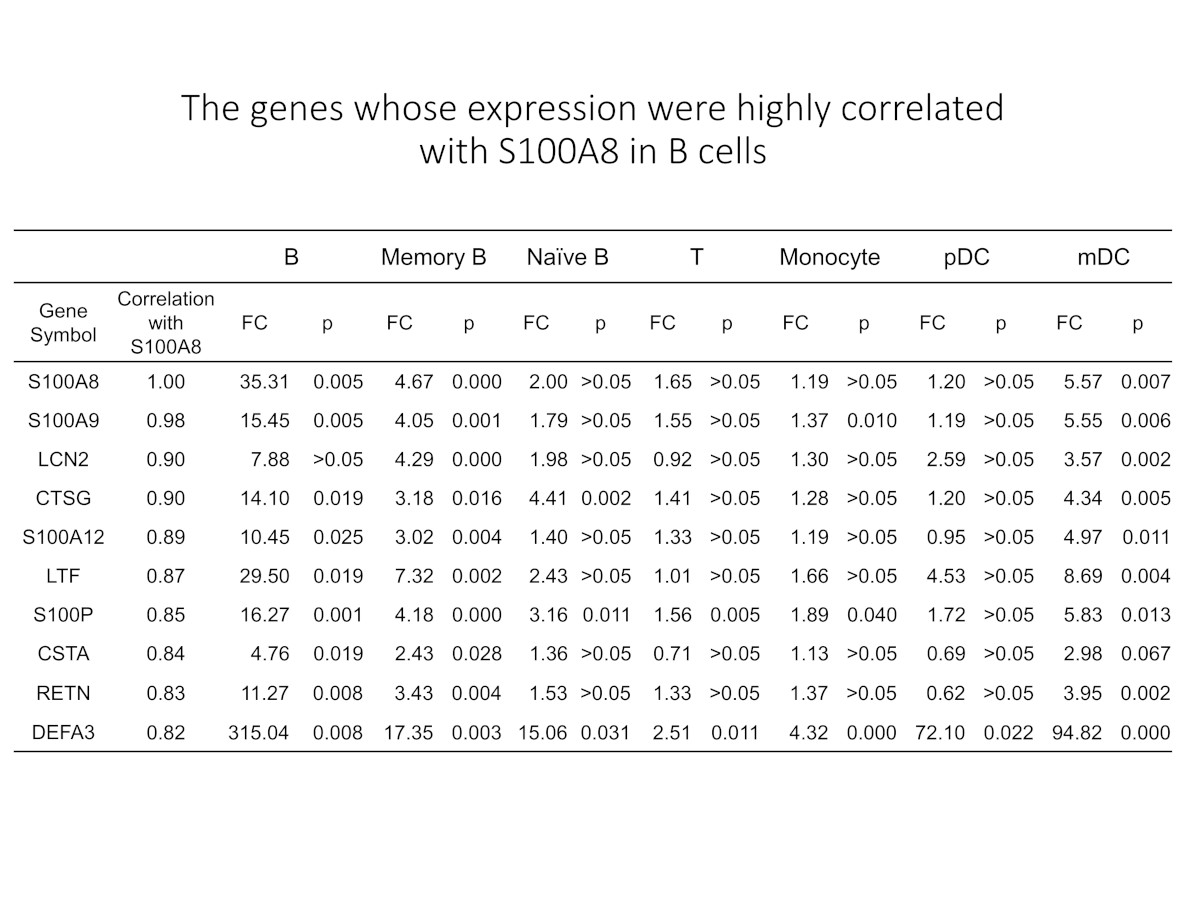

Supplement: Supplementary file 4 — Additional file 4: Supplementary Table 4. The genes whose expression were highly correlated with S100A8 in B cells. The genes whose expression were highly correlated with S100A8 in peripheral B cells were listed by Pearson’s correlation coefficients. The gene expression was analyzed by DNA microarray in the peripheral total B cells, memory B cells, Naïve B cells, T cells, monocytes, pDC, and mDC. Fold changes in the SLE patients compared with HC are shown. The listed molecules included many granulocyte-related molecules. The expression pattern in the total B cells was similar to those in memory B cells and mDC. FC: fold change, pDC: plasmacytoid dendritic cell, mDC: myeloid dendritic cell. [file 13075_2023_3057_MOESM4_ESM.jpg]

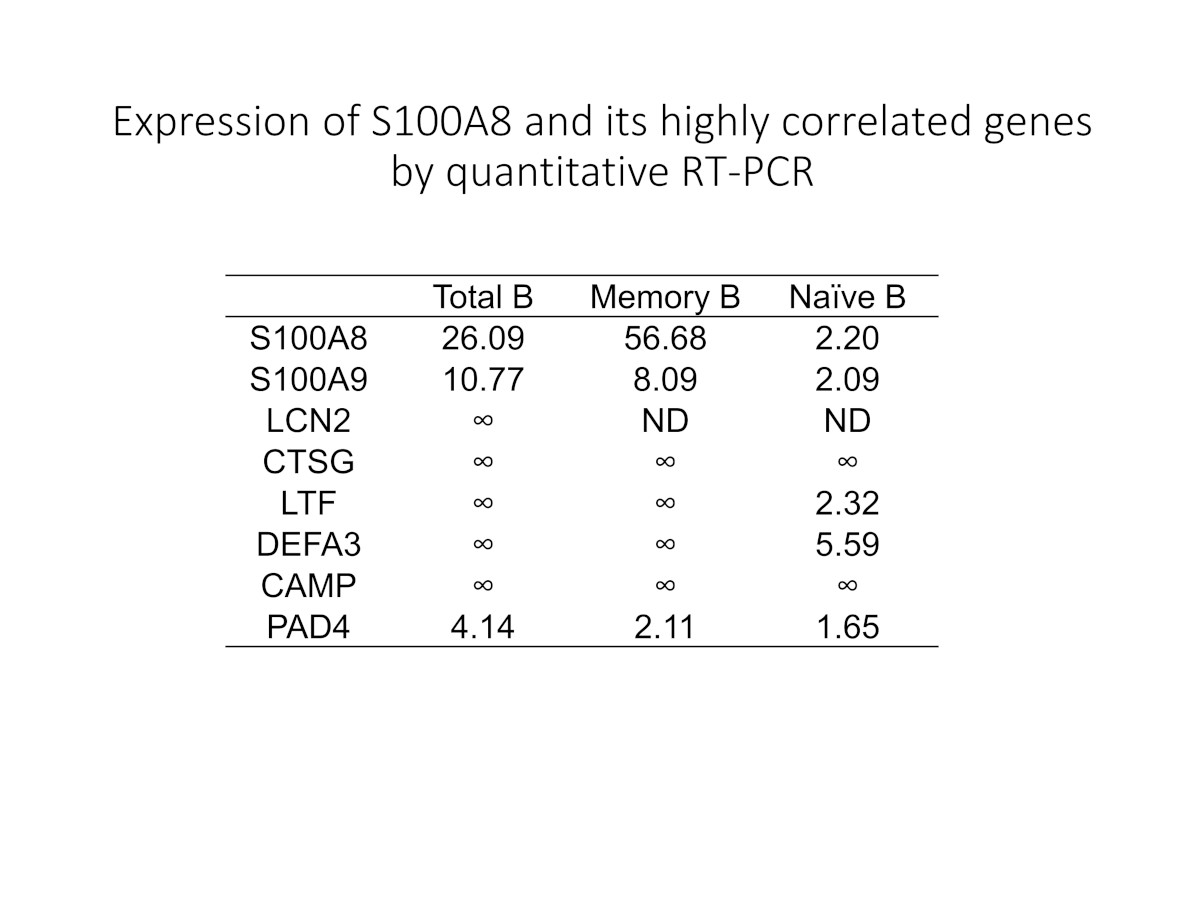

Supplement: Supplementary file 5 — Additional file 5: Supplementary Table 5. Expression of S100A8 and its highly correlated genes by quantitative RT-PCR. The expression of S100A8 and seven genes that showed a high correlation with S100A8 in DNA microarray were validated by quantitative RT-PCR in the peripheral total B cells, memory B cells, and naïve B cells. The values are fold changes (FC) in the SLE patients compared with HC. Memory B cells showed higher FCs than naïve B cells. ND: Not detected both in the SLEpatients and HC, ∞: Not detected in the HC. [file 13075_2023_3057_MOESM5_ESM.jpg]

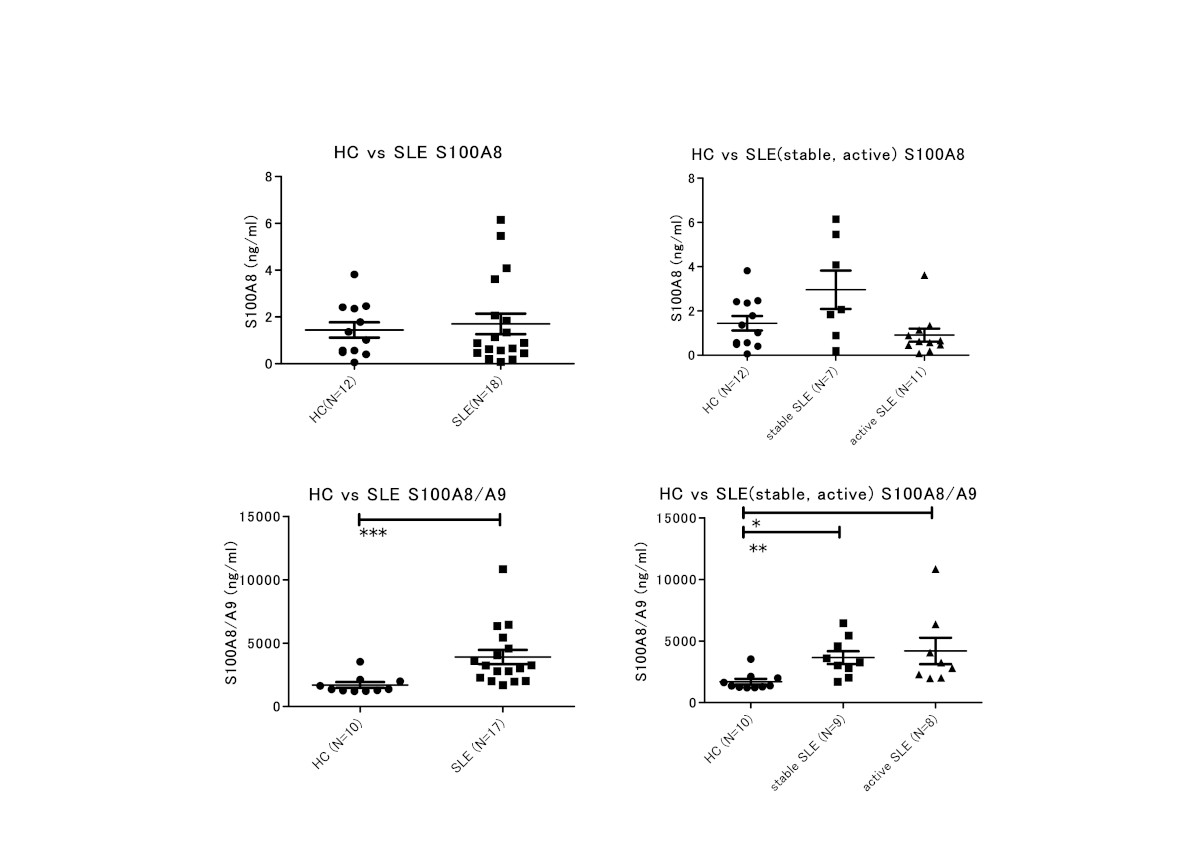

Supplement: Supplementary file 6 — Additional file 6: Supplementary Figure 1. Concentration of S100A8 and S100A8/A9 in plasma. Plasma S100A8/A9 concentration was significantly higher in SLE. Plasma S100A8 concentration was not significantly different between HC and SLE. Mann-Whitney U test as performed for comparison between two groups. Kruskal-Wallis test was performed for multiple comparison. *: p<0.05. **: p<0.01. [file 13075_2023_3057_MOESM6_ESM.jpg]

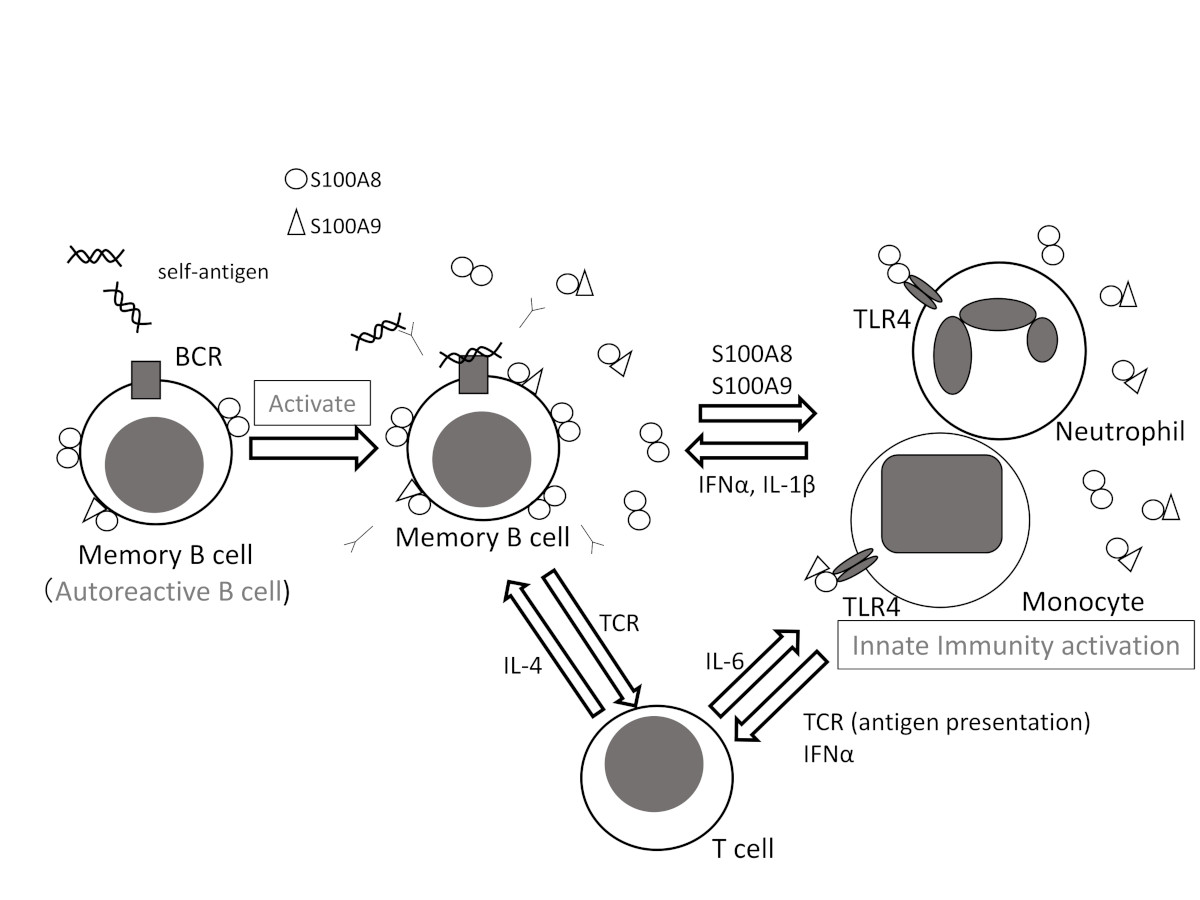

Supplement: Supplementary file 7 — Additional file 7: Supplementary Figure 2. Hypothetical schema of the involvement of S100A8 and S100A8/A9 in the pathophysiology of SLE. S100A8 and S100A8/A9 produced and secreted by memory B cells stimulates neutrophils and monocytes through TLR4. The innate immune cells activate acquired immune cells (T cells and B cells) by antigen presentation. Autoimmunity was promoted by this inflammatory cycle. [file 13075_2023_3057_MOESM7_ESM.jpg]

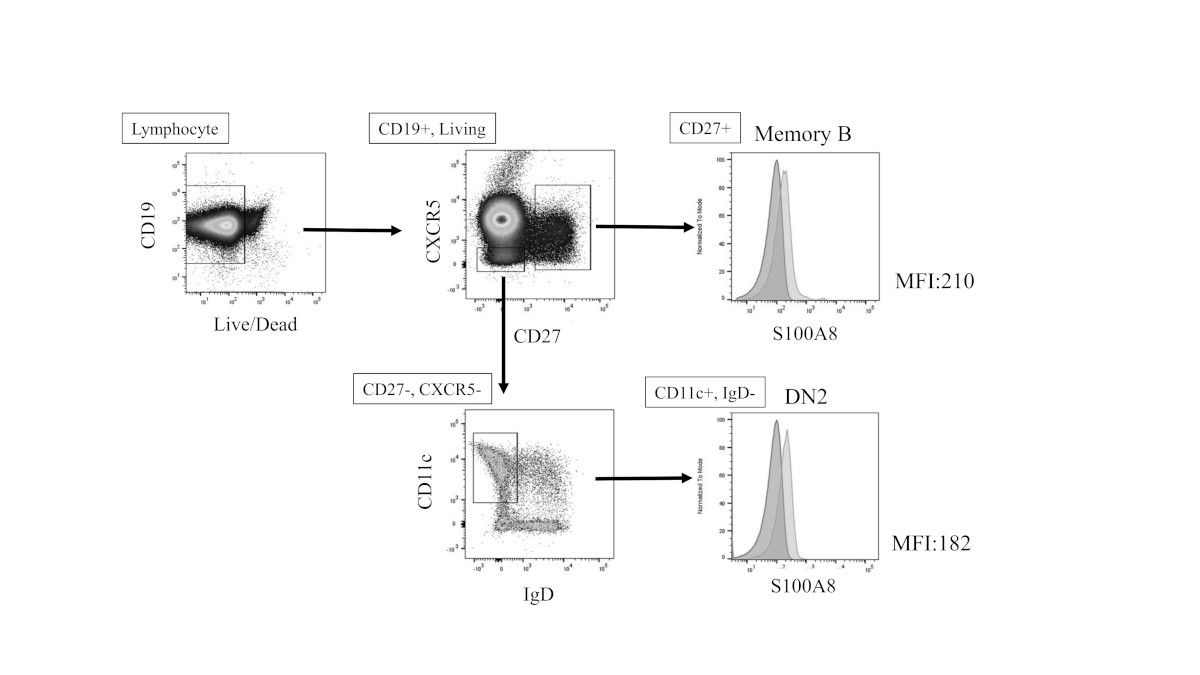

Supplement: Supplementary file 8 — Additional file 8: Supplementary Figure 3. Expression of S100A8 on DN2 B cells in SLE. In the peripheral blood of SLE patients, there was no significant change in the expression level of S100A8 on B cells between the memory B cell group and the DN2 B cell group. [file 13075_2023_3057_MOESM8_ESM.jpg]

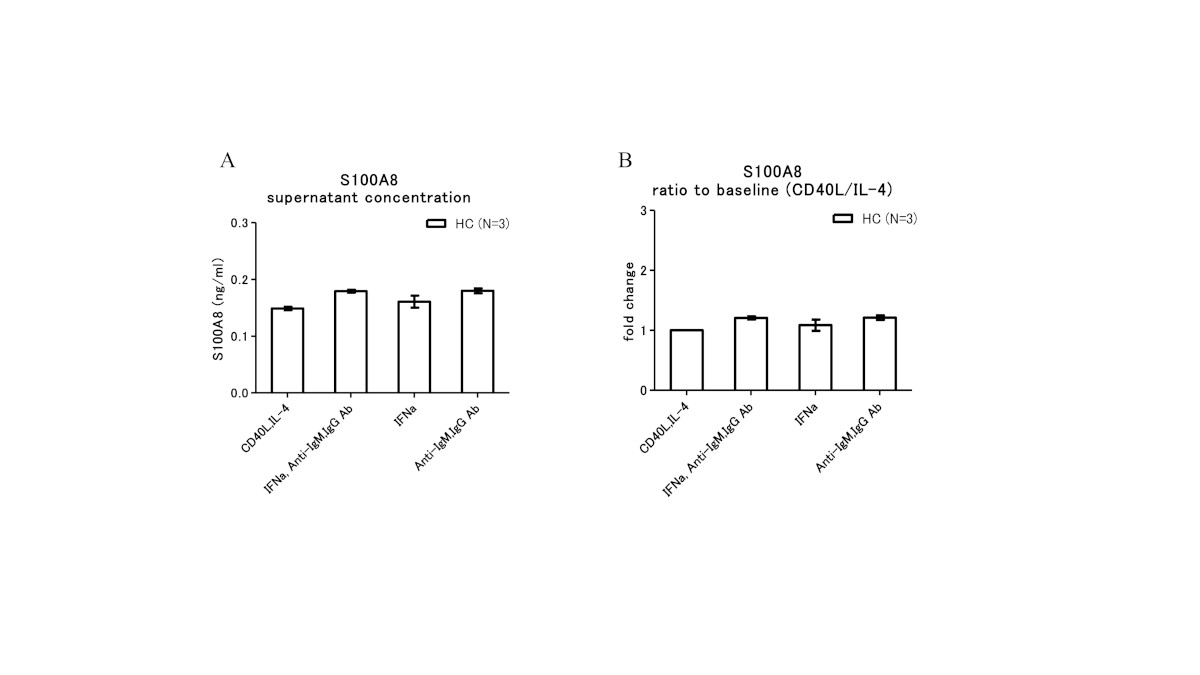

Supplement: Supplementary file 9 — Additional file 9: Supplementary Figure 4. Concentration of S100A8 in supernatant after stimulation on HC B cells with IFN-α and Anti-IgG/IgM. IFN-α and Anti-IgG/IgM were used to stimulate HC B cells. A. The supernatant concentration of S100A8 from the B cells of HC after the stimulation by IFN-α and/or Anti-IgG/IgM. The concentration of S100A8 showed no significant difference after the IFN-α andAnti-IgG/IgM stimulation. B. The ratios of the concentration to the baseline (CD40L and IL-4 only) are indicated. The ratio of S100A8 from the B cells of HC was not elevated after the stimulation. [file 13075_2023_3057_MOESM9_ESM.jpg]
